# The need for independent research on the health effects of glyphosate-based herbicides

**DOI:** 10.1186/s12940-018-0392-z

**Published:** 2018-05-29

**Authors:** Philip J. Landrigan, Fiorella Belpoggi

**Affiliations:** 1 Environmental Medicine and Pediatrics Arnhold Institute for Global Health Icahn School of Medicine at Mount Sinai, One Gustave L. Levy Place, Box 1057, New York, NY 10029 USA; 2 Cesare Maltoni Cancer Research Center Ramazzini Institute, Via Saliceto, 3, 40010 Bentivoglio, Bologna Italy

**Keywords:** Glyphosate, Roundup, Glyphosate based herbicides, GBH, Carcinogenicity, Crowdfunding

## Abstract

**Background:**

Glyphosate, formulated as Roundup, is the world’s most widely used herbicide. Glyphosate is used extensively on genetically modified (GM) food crops designed to tolerate the herbicide, and global use is increasing rapidly. Two recent reviews of glyphosate’s health hazards report conflicting results. An independent review by the International Agency for Research on Cancer (IARC) found that glyphosate is a “probable human carcinogen”. A review by the European Food Safety Agency (EFSA) found no evidence of carcinogenic hazard. These differing findings have produced regulatory uncertainty.

**Regulatory actions:**

Reflecting this regulatory uncertainty, the European Commission on November 27 2017, extended authorization for glyphosate for another 5 years, while the European Parliament opposed this decision and issued a call that pesticide approvals be based on peer-reviewed studies by independent scientists rather than on the current system that relies on proprietary industry studies.

**Ramazzini Institute response:**

The Ramazzini Institute has initiated a pilot study of glyphosate’s health hazards that will be followed by an integrated experimental research project. This evaluation will be independent of industry support and entirely sponsored by worldwide crowdfunding. The aim of the Ramazzini Institute project is to explore comprehensively the effects of exposures to glyphosate-based herbicides at current real-world levels on several toxicological endpoints, including carcinogenicity, long-term toxicity, neurotoxicity, endocrine disrupting effects, prenatal developmental toxicity, the microbiome and multi-generational effects.

## Background

### History and use

Glyphosate is the world’s most widely used herbicide [1]. Glyphosate Based Herbicides (GBHs) were first authorised for agricultural use in the US in 1974 by the Environmental Protection Agency. In Europe, glyphosate was authorised by the European Commission in 2002. In the US, glyphosate use has increased by more than 250-fold in the past 4 decades — from 0.4 million kg in 1974 to 113 million kg in 2014. Global glyphosate use has also increased from 3200 tons/year in 1974 to 825,000 tons/year in 2014, and glyphosate is now used in over 140 countries [1]. In future years, glyphosate use is projected to continue to increase and by 2020 is estimated to reach one million tons per year.

Glyphosate, formulated as Roundup, is used on corn and soybeans that have been genetically engineered to be resistant to glyphosate. These “Roundup- Ready” crops were first introduced in the mid-1990s and now account for more than 90% of the corn and soybeans planted in the United States [2]. Today glyphosate is contained in over 750 commercial herbicide products designed for intensive crop-growing, market gardening and gardens in general. This massive use of glyphosate in the most varied sectors of agriculture has led to widespread environmental dissemination. Trace levels of glyphosate can now be found widely in soil, foodstuffs, air and water as well as human urine [3–5].

### Regulatory actions

On November 27 2017, the European Commission extended the authorization for glyphosate for another 5 years. The European Parliament, however, opposed this decision and issued a call for pesticide approvals to be based on published peer-reviewed studies by independent scientists instead of the current system, which is largely based on unpublished proprietary studies. Regulatory uncertainty and debate are extensive [6, 7]. Key milestones in the risk assessment process that has led to the current regulatory debate about the safety of glyphosate may be summarized as follows:

- March 2015: the World Health Organization’s International Agency for Research on Cancer (IARC) conducted an extensive review of the published peer-reviewed epidemiologic, toxicologic and genetic literature on glyphosate, independent of influence by the pesticide manufacturing industry, and concluded that glyphosate is “probably carcinogenic to man” (Category 2A [8]).
- November 2015: the EFSA deemed glyphosate “unlikely to pose a cancer risk for man”. That conclusion was based on a glyphosate renewal assessment report (RAR) presented in January 2014 by the Federal German Institute for Risk Assessment (Bundesinstitut für Risikobewertung, BfR) [9]. The EFSA and RAR review groups included scientists that did not disclose their names and financial interests and also relied on unpublished, non-peer-reviewed reports generated by industry [10].
- March 2017: following a heated argument over the safety of glyphosate, and numerous deferments of the European ballot, the European Union (EU) appointed the European Chemicals Agency (ECHA) to look into the issue of glyphosate toxicity. The ECHA’s Risk Assessment Committee analysed an enormous amount of scientific data and concluded that “the scientific evidence so far available does not satisfy the criteria for classifying glyphosate as carcinogenic, mutagenic or toxic for reproduction.” [11]. According to the ECHA, glyphosate may cause grave damage to the eyes and be toxic to aquatic organisms with long-term effects.
- November 2017: The EU voted to extend glyphosate authorization for an abbreviated period of five years; the Acceptable Daily Intake (ADI) was increased from 0.3 to 0.5 mg/kg bw/day [12]. The deliberation frustrated parties on all sides. Agrochemical companies criticized the review process as driven more by politics than science after it became clear that the weed killer’s use would not be re-authorized for the 15 years typical for such chemicals. Environmental advocates said that the agrochemical industry had tainted scientific reviews in Europe by interfering in them.

## Main text

### The Ramazzini Institute research project

#### Pilot study

A ‘pilot’ experimental study of the toxicity of GBHs was carried out at the Ramazzini Institute in 2016 (Ministerial Authorization N° 710/2015-PR, issued on 17/7/2015) where both glyphosate alone and its formulation Roundup have been tested. In fact glyphosate alone and its formulations could have different effects. For example, the adjuvants present in the formulation might potentiate the toxic effects of glyphosate [13]. To set this study in motion, the Institute built up a network of authoritative partners including the University of Bologna (Departments of Agriculture, Veterinary Science and Biostatistics), the Genoa Istituto Tumori, the Istituto Superiore di Sanità (ISS), the Icahn School of Medicine at Mount Sinai, New York, and the George Washington University, Washington, DC.

The study was designed to assess the techniques and methods for detecting glyphosate and its metabolites in different matrices [14] and to develop methods for assessing organ toxicity, genotoxicity, molecular toxicity, reproductive/developmental toxicity, endocrine disruption and microbiome alteration [15]. In this pilot study, glyphosate and Roundup were both tested at a dose considered to be “safe”- corresponding to the ADI of glyphosate currently allowed in the US, defined as the chronic Reference Dose (cRfD) determined by the US EPA [16], namely 1.75 mg/kg bw/day.

Initial results from this pilot study were presented during the Annual Ramazzini Days (26–29 October 2017). These preliminary findings suggest that glyphosate and Roundup – even at doses deemed safe, i.e., at doses equivalent to the current ADI and with relatively short exposure time, from pregnancy until 13 weeks after weaning in human-equivalent terms from pregnancy to approximately 18 years of age – might be able to alter certain important biological parameters related to sexual development, genotoxicity and alteration of the intestinal bacterial flora. Other important parameters are under investigation that pertain to effects on target organs such as mammary gland, kidney and liver, the hormonal status in the blood, and chromosome alterations in sperm. All the results will be submitted for publication in this journal [14, 15].

A pilot study is, by definition, of short duration and involves fewer animals than a comprehensive experiment. Therefore, it can provide only limited information and is not designed to detect chronic effects and diseases of late onset such as cancer. Thus the Ramazzini Institute pilot study is not able to resolve the current regulatory uncertainty around glyphosate. However, the findings of the pilot study do highlight potentially serious health effects that might manifest as long-term oncologic pathology and could affect very large numbers of people, given the great and growing global use of the GBHs. Clearly these findings deserve further follow-up.

#### Future research

To follow up on the Ramazzini Institute pilot study, a more comprehensive investigation is necessary and it must examine the effects of a range of different environmentally relevant doses of glyphosate alone and GBHs. Therefore, in 2015, the Ramazzini Institute designed a comprehensive, integrated experimental approach to a long-term project following an already published protocol through which numerous parameters bearing on human health might be simultaneously monitored, thereby sparing animals [17]. In fact, proprietary studies conducted on behalf of the manufacturers often represent a limited investigation of the various toxicological effects now studied by academic and government scientists. The integrated study proposed by the Ramazzini Institute is based on a stepwise process that includes the priority end points of the Economic Co-operation and Development and the National Toxicology Program guidelines on carcinogenicity and chronic toxicity in addition to developmental and reproductive toxicity, exploring multiple windows of susceptibility of specific interest for risk assessments and public health decision-making such as prenatal, lactational and neonatal exposures. Such an integrated toxicological study is needed, together with further epidemiological evidence, for an independent and comprehensive assessment of the possible risks resulting from the ubiquitous exposure to GBHs.

As in the pilot study, both glyphosate and the commercial formulation Roundup will be tested in the integrated study. A human-equivalent model will be used to determine the dose-levels to be administered and the exposure period, which will include mating and gestation. Detailed assessments will examine the toxic effects in terms of the intestinal microbiome, gene expression and parameters relating to fertility, defects in development, effects on the nervous system and any treatment-related differences in the incidence of various tumours. This will be the most comprehensive study on GBHs to date and it will last 3–4 years.

To preserve independence from the pesticide-manufacturing industry and from its competitor (i.e. organic food industry), this integrated study will be supported through a global crowd-funding campaign that will be open to the world’s citizens, non-governmental organizations (NGOs) and national/international institutions. Details of this campaign are available at: www.glyphosatestudy.org.

To provide ongoing review of the integrated study, we intend to set up an external international scientific committee that will evaluate the study plan, the conduct of the study and review study results as they become available. We also plan to gather together all stakeholders interested in using our results to ascertain the degree of hazard involved in GBH exposure. These will include: IARC, EFSA, ISS, the National Institute of Environmental Health Sciences, and others, including NGOs representatives. Study results will be available by the time of the next EU decision on the reauthorization of glyphosate in 2022.

## Conclusions

Whatever the outcome of the Ramazzini Institute study, the findings will provide regulatory agencies and policy-makers with solid independent results obtained by a shared research project on which they can confidently base their risk assessments and their evaluations, including the upcoming decision for the reauthorization for glyphosate use in Europe in 2022.

## Abbreviations

ECHA: European Chemicals Agency
EFSA: European Food Safety Agency
EU: European Union
GBH: Glyphosate Based Herbicides
GM: Genetically modified
IARC: International Agency for Research on Cancer
ISS: Istituto Superiore di Sanità
RAR: Renewal assessment report
